# Platelet-Rich Fibrin-Enhanced Bone Healing Co-grafted With Either Hydroxyapatite and Beta Tricalcium Phosphate or Demineralized Freeze-Dried Bone in Small Maxillofacial Osseous Defects: A Clinical Comparison

**DOI:** 10.7759/cureus.44048

**Published:** 2023-08-24

**Authors:** Arshad Khan, Iqbal Ali, Harsha Pradhan, Sameera Shamim Khan, Ashish Tripathi, Rifaquat Ali

**Affiliations:** 1 Oral and Maxillofacial Surgery, Career Post Graduate Institute of Dental Sciences & Hospital, Lucknow, IND; 2 Oral and Maxillofacial Pathology and Oral Microbiology, Career Post Graduate Institute of Dental Sciences & Hospital, Lucknow, IND; 3 Oral and Maxillofacial Surgery, Private Practice, Ghazipur, IND

**Keywords:** beta tricalcium phosphate, demineralized freeze-dried bone, platelet-rich fibrin, periapical lesion, hydroxyapatite

## Abstract

Introduction: This study was conducted to clinically compare a commercially available combination of 70:30 nanocrystalline hydroxyapatite (HA) and beta tricalcium phosphate (βTCP) along with platelet-rich fibrin (PRF) with demineralized freeze-dried bone (DFDB) grafts along with PRF in small maxillofacial osseous defects.

Materials and methods: Thirty patients with one osseous defect were randomly distributed into two groups of 15 each: Group A and Group B. Group A patients received HA+βTCP+PRF while Group B received DFDB + PRF. Postoperative pain, swelling, wound dehiscence, and the presence or absence of infection were evaluated at various intervals up to seven postop days and compared between the two groups and within either group. A technetium 99m methylene diphosphonate (MDP ^99m^Tc) scan was also done for a representative patient of either group at the end of three months to evaluate the fate of the graft.

Results: We found no significant difference between the two groups for any of our parameters. Significant improvements were noted for pain and swelling within either group at various intervals. The MDP ^99m^Tc scan showed increased tracer uptake for the representing patient of either group.

Conclusions: HA+βTCP is more inexpensive than DFDB and more readily available and has no host incompatibility or infection potential, resulting in similar clinical postoperative states as DFDB when either is used with PRF.

## Introduction

We conducted this study to clinically compare a commercially available combination of 70:30 nanocrystalline hydroxyapatite (HA) and beta tricalcium phosphate (βTCP) along with platelet-rich fibrin (PRF) with demineralized freeze-dried bone (DFDB) grafts along with PRF in small maxillofacial osseous defects.

The bone growth phenomenon factored by bone morphogenetic proteins is an example of osteoinduction which is defined as “the process by which exogenous growth factors promote differentiation of host mesenchymal stem cells to form chondroblasts and osteoblasts that form new bone” [1]. Seen frequently in bone healing, it is inclusive of the recruitment of immature cells and their stimulation to develop into proosteoblasts [2]. On the other hand, osteoconduction is defined as “the process by which an implanted scaffold passively allows ingrowth of host vasculature, cells, and tissue” [1]. Although interrelated, these are not identical [2]. Besides being osteoconductive, a demineralized bone matrix is strongly osteoinductive [1]. Tricalcium phosphate is osteoconductive but not osteoinductive [1]. HA is known to be osteoconductive [3]. Although possibly host incompatible and capable of infection transmission [4] (besides being relatively expensive and difficult to procure as compared to either HA or βTCP) DFDB, being osteoinductive, is a superior graft clinically [5,6]. We investigated whether or not HA + βTCP comes to a clinical par with DFDB when either is used with PRF. Thus, the hypothesis we studied was that when used with PRF, the advantages of using DFDB get eclipsed by the PRF and thus HA+βTCP is a more suitable graft material when used with PRF because of the advantages of this combination. PRF enhances bone healing [7] by providing a sustained release of endogenous fibrogenic factors which are important for wound healing [8] and causes a rapid reduction in pain [7] and swelling [7,9]. HA [10], βTCP [11], and DFDB [12] have been combined with PRF independently for use in osseous defects to achieve faster bone regeneration and repair. Being a dynamic bone scanning modality that provides functional information is sensitive to subtle changes in bone perfusion and bone turnover, and provides highly sensitive, three-dimensional imaging of the skeleton, 99mTc methyl diphosphonate scanning provides a quantitative measurement of bone blood flow and regeneration after surgical intervention [13].

## Materials and methods

Ethical clearance was obtained from our institutional ethics committee before the commencement of the study. The study was designed to compare pain, swelling, infection, and wound dehiscence between two groups of 15 patients each. This sample size was chosen as we were undertaking a purely clinical study. We did not consider allocation concealment to be of relevance to our study. Thirty patients with a periapical lesion in either jaw, appearing more than 5mm but less than 20mm approximately at the largest diameter (estimated by unaided eyes, on a radiograph) who did not need extraction, came to the O.P.D. of the Dept. of Oral & Maxillofacial Surgery of Career Post Graduate Institute of Dental Sciences & Hospital, and were otherwise healthy were included. Preoperative assessment comprised a detailed medical and dental history, clinical and radiographic evaluation, and hematological investigations to establish general health. Patients whose platelet count was less than 150000 per mm^3^ were excluded, as were patients with any systemic disease, compromised immunity, pregnant women, and lactating mothers. Cases were selected irrespective of age, sex, caste, religion, and socioeconomic status. After this, written informed consent was obtained and the 30 patients included in the study were randomly assigned to one of the two groups: Group A included patients with a periapical bone defect receiving HA+βTCP along with PRF and Group B included patients with a periapical bone defect receiving the DFDB allograft along with PRF. No particular procedure for randomization was followed.

Oral prophylaxis for all patients was done before surgery (at the time when hematological examination results were awaited, typically one day before surgery). The HA+βTCP graft material by the brand name Sybograf by Eucare Pharmaceuticals Pvt. Ltd. in the HA: βTCP ratio of 70:30 was used. DFDB (500 to 1000μ m particle size) was procured from Tata Memorial Hospital Tissue Bank, Mumbai.

Method of preparation of PRF

Fifteen milliliters of whole venous blood was collected from every patient and divided equally into two sterile, 10ml, Borosil test tubes (without an anticoagulant) and placed into opposing slots of an electric swing out centrifuge of the brand Remi. Centrifuging these at 3000 revolutions per minute (rpm) for 10 mins resulted in blood settling into three layers of which PRF, the middle layer [14], was obtained from both test tubes. The remaining two layers were discarded. The PRF from one of the test tubes was then placed between two sterile surfaces and pressured to get a flat membrane, the PRF membrane [15].

Surgical procedure

After the administration of 2% lignocaine with 1:200,000 adrenaline to achieve local anesthesia, the standard surgical protocol for removal of the pathology and curettage was followed. The tooth was endodontically treated. Packing the defect with the HA+βTCP bone graft with PRF in Group A patients and DFDB along with PRF in Group B patients was done next. Freshly prepared PRF was mixed with HA+βTCP or DFDB bone graft by sprinkling the particulate graft material over the PRF gel and together the mixture was placed into the postcurettage intrabony defect making sure that the cavity was tightly packed with the graft material. The window opening of the cavity was covered with the PRF membrane to ensure the stability of the graft material. The mucoperiosteal flap was placed over the membrane and sutured watertight by interrupted 3 (0) black silk sutures.

On the seventh postoperative day, sutures were removed. All patients of either group were prescribed amoxicillin 500mg combined with potassium clavulanate 125 mg thrice daily and metronidazole 400mg thrice daily along with ketorolac tromethamine twice daily for five days. Mouth rinsing with 0.2% w/v chlorhexidine gluconate (Clohex) was advised twice daily for 15 days.

Postoperative follow up

All patients were evaluated clinically for postoperative pain using a visual analog scale (VAS) of 0 to 10 with 0 being no pain and 10 being the worst imaginable pain on the first, second, third, fifth, and seventh postop days. Swelling, dehiscence, and infection were evaluated clinically for the presence or absence on the first, third, and seventh postop days. An MDP 99mTc scan was done in the third postop month for one representative patient of either group. 

Data obtained were compiled into a master chart and statistically analyzed using IBM SPSS Statistics for Windows, Version 23 (Released 2015; IBM Corp., Armonk, New York, United States).

Statistical analysis comprised mean and standard deviation, the chi-square test, and the Mann Whitney ‘U’ test of thus-obtained data. A P value less than 0.05 was considered significant.

## Results

Group A patients had a mean age of 30.80 years ±11.87 years ranging between 15 and 60 years. For Group B patients, the mean age was 30.20 years ± 17.29 years ranging between 15 and 55 years; there was no significant difference between the two groups for age. Eight patients of Group A were females and seven were males. Among Group B patients, six were females and nine were males. Overall, the study comprised 14 females and 16 males; there was no significant difference between the two groups as far as the female: male ratio was concerned.

**Figure 1:**
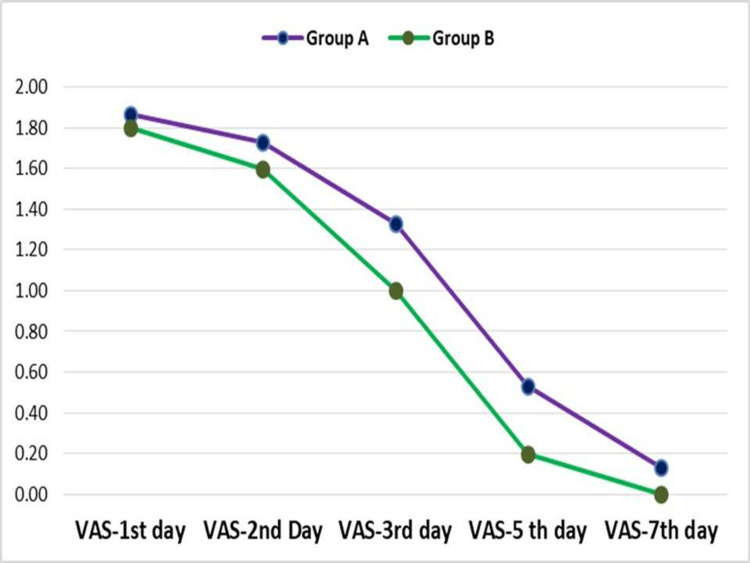
Intergroup Comparison of VAS Pain Scores VAS: Visual Analog Scale

**Figure 2:**
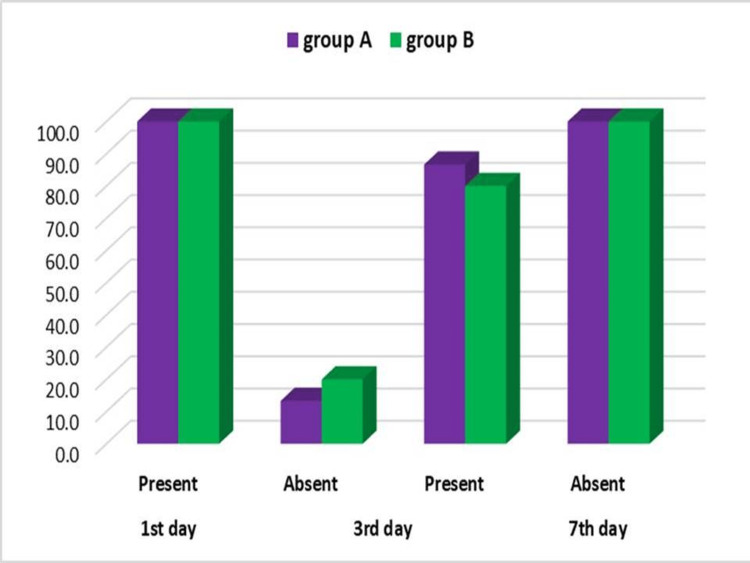
Intergroup Comparison of Swelling

**Figure 3:**
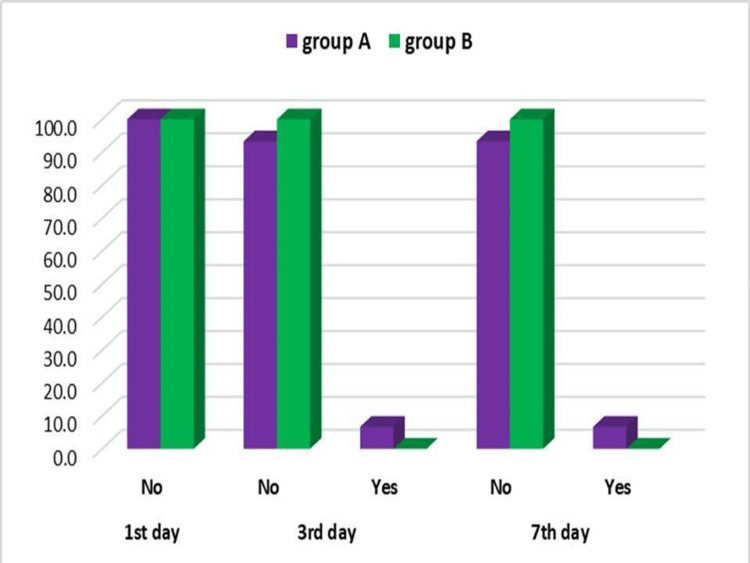
Intergroup Comparison of Infection and Dehiscence

Despite being prescribed an analgesic, patients had pain and swelling. On comparing the VAS pain score (Figure 1), swelling (Figure 2), infection, and dehiscence (Figure 3) between the two groups, we found no significant difference for any of these parameters at any stage of observation.

Within both groups, significant decreases in VAS scores were noted between the first day and third day, first day and fifth day, first day and seventh day. Between the second day and third day, second day and fifth day, second day and seventh day, third day and fifth day and third day and seventh day too, the reduction in VAS scores was significant in either group. Between the fifth day and seventh day, the drop in pain was significant in group A and insignificant in group B. These observations suggest a rapid, sustained drop in pain in both groups (Figure 1). 

**Figure 4:**
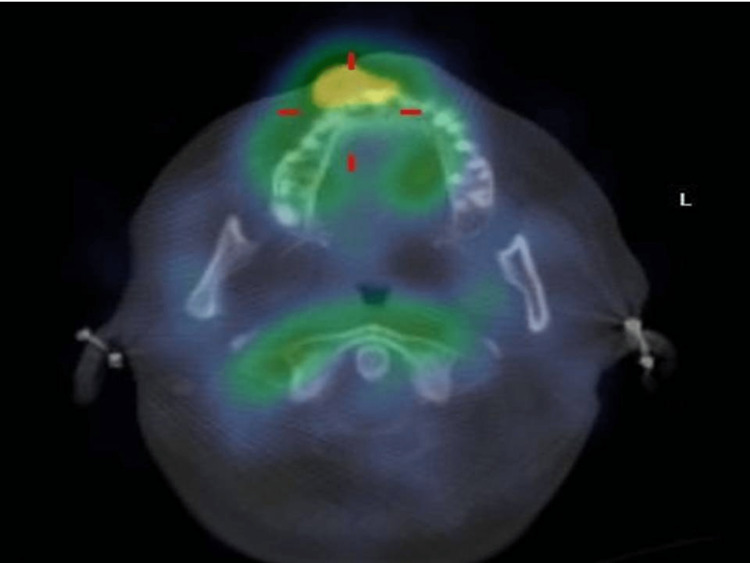
Technetium Scan of a Group A Patient

**Figure 5:**
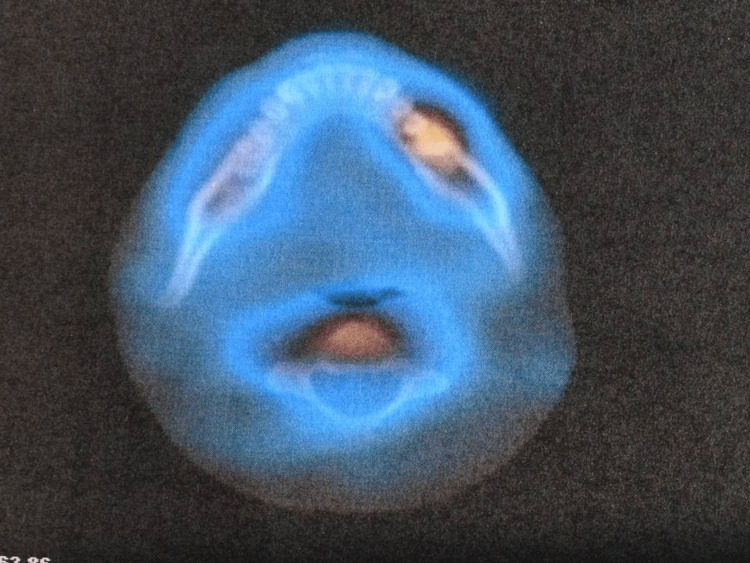
Technetium Scan of a Group B Patient

On comparing wound dehiscence and infection data between the two groups (at postop days 1, 3, and 7), we found no significant difference between the two groups at any stage even though one patient of group A did show signs of both infection and wound dehiscence on postop day 3. This patient had been treated at the same site a year before the study. This treatment met with failure and recurrence of the lesion. The subsequent lesion was larger than other lesions of the study and thus carried a greater risk of failure. Further treatment for this patient was carried out separate from this study. The 99mTc MDP bone scan showed increased uptake of tracer at the graft site in the representing patient of either group suggesting the viability of the graft in both groups (Figures 4, 5 ). 

## Discussion

Although DFDB, being expensive, not as easy to obtain as HA+βTCP, possibly host incompatible [4], and carrying with it a risk of infection [4], is not the ideal graft material, it does have the advantage (over both HA & βTCP) of being osteoinductive and hence clinically superior [5,6]. HA and βTCP, being synthetic [16], have neither host incompatibility issues nor carry a risk of infection. Also, these are relatively inexpensive and easy to obtain. Yet, their disadvantage, that either is simply osteoconductive not only disqualifies these as ideal graft materials but also renders these clinically inferior to the DFDB allograft [5,6] despite their advantages. Enhancement of the HA [17] and βTCP [18] graft materials themselves has been attempted. PRF not only enhances nonvital graft material quality, but also enhances bone healing [7]. If PRF enhancement of HA+βTCP graft defect healing would result in clinically superior healing or even if it would be at par with PRF-enhanced DFDB graft defect healing, the advantages of HA+βTCP would make it the superior particulate graft material. From a clinical viewpoint, after PRF healing enhancement, the role of the particulate graft material would remain to provide body to the filling material. Singh et al. observed that autologous PRF is biocompatible and causes significantly improved soft and hard tissue healing in extraction sockets [19].

As far as our observations regarding VAS scores for pain are concerned, we found no significant difference between the two groups at any stage. This was suggestive of the equivalence of either particulate graft material we used as far as pain is concerned. Within either group, the reduction in VAS scores was significant at various intervals pointing at the minimally traumatic and sterile nature of our surgical technique besides the healing-enhancing activity of PRF. Choukroun et al. also found a rapid reduction in patient discomfort following the use of PRF [7]. Singh et al., who did not use a bone graft, also reported a decrease in pain as compared to contralateral, no PRF third molar extraction sites in a split-mouth study [19].

On comparing postop swelling on the first, third, and seventh postop days, we found no significant difference for this parameter between the two groups at any of these stages. Since both groups improved similarly, we reasoned that both the particulate grafts we used are similar as far as improvement in postoperative swelling is concerned. On day 1, all patients of both groups had swelling, which is a physiological response to surgery and a contribution to this was possibly made by the watertight closure of the surgical site which is mandatory after every graft procedure. On day 3, most patients still had swelling while some did not. We attribute this to differential surgical trauma depending upon the site of the lesion and individual variation in response to surgery. By the seventh postop day, no patient of either group had swelling, pointing to the minimal relevance of these factors. We explain this widespread improvement in swelling by the seventh postop day across both groups by the presence of PRF which discourages swelling [7,9]. The growth factors found in PRF are mitogenic, chemotactic, and angiogenic [7]. PRF membranes provide a favorable physiological architecture to support the healing process and result in a decreased inflammatory response [7]. Del Fabbro et al. stated that the use of platelet concentrates is related to a lower level of swelling and pain [9]. 

On comparing wound dehiscence and infection data gathered on the first, third, and seventh postop days between the two groups, we found no significant difference. Yet again, the similarity, clinically, between either particulate graft, when used with PRF was suggested. It was only on the third day that one patient of group A showed wound dehiscence and signs of infection. This patient had undergone treatment for the same problem one year before with grafting of the same site and this met with failure and recurrence of the lesion. The subsequent lesion was larger than other lesions of the study and thus carried a greater risk of failure. Further treatment for this patient was separate from this study. 

At the end of the third month follow up, the representing patient of either group showed increased uptake of tracer at the bone graft site in the 99mTc MDP bone scan which is suggestive of the viability of their grafts. This can be explained by a statement made by Lundquist et al who evaluated the bioactivity and stability of endogenous fibrogenic factors in PRF wherein they stated that PRF provides sustained release of endogenous fibrogenic factors important for wound healing and protection against their proteolytic degradation [8]. 

Since our study did not have the benefit of external funding, it is limited by a small sample size. Studies of a larger sample size are needed to corroborate or refute these findings. 

## Conclusions

To conclude, the following inferences can be made: PRF accelerates wound healing and reduction in discomfort to similar levels for HA+βTCP and DFDB. The HA+βTCP combination has several advantages over DFDB: an abundant supply of material, higher cost efficiency, and the absence of the risk of transmission of disease or host incompatibility potential. Both HA+βTCP and DFDB can be used with or without PRF in small maxillofacial osseous defects. The addition of PRF to DFDB and HA+βTCP improves the handling characteristic of the graft making it easier to uniformly pack in the defect with minimum graft material loss. PRF prevents loss and failure of bone graft, which is possible given the highly dynamic environment of the oral cavity.
